# The associations between psychosocial working conditions and changes in common mental disorders: a follow-up study

**DOI:** 10.1186/1471-2458-14-588

**Published:** 2014-06-11

**Authors:** Hanna Laine, Peppiina Saastamoinen, Jouni Lahti, Ossi Rahkonen, Eero Lahelma

**Affiliations:** 1 Department of Social Research, University of Finland, PL 54, 00014 Helsinki, Finland; 2 Hjelt Institute, Department of Public Health, University of Helsinki, PL 41, 00014 Helsinki, Finland; 3 Finnish Institute of Occupational Health, Topeliuksenkatu 41 a, 00250 Helsinki, Finland

**Keywords:** Common mental disorders, GHQ-12, Changes, Psychosocial working conditions, Follow-up

## Abstract

**Background:**

Common mental disorders (CMD) are prevalent in working populations and have adverse consequences for employee well-being and work ability, even leading to early retirement. Several studies report associations between psychosocial working conditions and CMD. However, there is a lack of longitudinal research within a broad framework of psychosocial working conditions and improvement in CMD. The aim of this study was to examine the associations between several psychosocial working conditions and deteriorating and improving CMD among ageing employees over a five-to-six-year follow-up period.

**Methods:**

The study is based on the Helsinki Health Study baseline survey in 2001–2002 and a follow-up in 2007 (*N* = 4340, response rate 83%) conducted among 40-60-year-old female and male employees. The General Health Questionnaire (GHQ-12) was used to measure common mental disorders. Psychosocial working conditions were measured in terms of job strain, organisational justice, work-family interface, social support and workplace bullying. The covariates included sociodemographic and health factors.

**Results:**

Following adjustment for all the covariates, family-to-work (OR 1.41, 95% Cl 1.04-1.91) and work-to-family conflicts (OR 1.99, 95% Cl 1.42-2.78) and workplace bullying (OR 1.40, 95% Cl 1.09-1.79) were associated with deterioration, and family-to-work conflicts (OR 1.65, 95% Cl 1.66-2.34) and social support (OR 1.47, 95% Cl 1.07-2.00) with improvement in CMD.

**Conclusions:**

Adverse psychosocial working conditions contribute to poor mental health among employees. Preventing workplace bullying, promoting social support and achieving a better balance between work and family may help employees to maintain their mental health.

## Background

Common mental disorders (CMD), defined as minor, non-psychotic mental-health problems [1], are prevalent in working populations and have adverse consequences for employee wellbeing and work ability [2]. On an annual basis, mental disorders affect one quarter of the population of Europe, increasing sickness absence and causing early exit from work due to decreased work ability [3,4]. In the case of Finland, mental-health problems are the leading cause of disability retirement [5].

From the perspective of prevention, it is important to consider the factors affecting mental health [6]. Working environments are everyday settings in which people spend much of their daytime. Psychosocial stress factors could be defined as aspects of work (e.g., content, organisation or social relations) that promote stress conditions characterised by symptoms or impaired functioning [7]. These factors also affect the way people perceive stress, their coping skills, and how adaptively they cope with the stressors of working life [8].

Previous empirical studies report associations between psychosocial working conditions and CMD, such as self-reported symptoms of depression and anxiety, mental problems based on clinical interviews, and diagnosed mental disorders [7,9,10]. The focus tends to be on environmental factors such as job strain and its consequences for health and functioning. Recent studies also include person-centred factors such as workplace bullying and work-family conflicts [11,12]. However, psychosocial factors are often examined separately or combined according to a certain model or theory. There is ongoing interest in integrating different models and thereby contributing to a more comprehensive understanding of a broad range of psychosocial working conditions that may affect mental health [13].

The aim of this study was to examine the associations between environmental and person-centred psychosocial working conditions and CMD. We measured CMD by means of the General Health Questionnaire (GHQ), which is a well validated [14,15], commonly used instrument for screening depression and anxiety disorders in population samples [16]. The GHQ also captures minor mental disorders [16], and could therefore be considered suitable for preventive purposes. The following review of empirical studies focuses on studies using the GHQ as a measure of CMD.

Cross-sectional studies report associations between common mental disorders and various psychosocial working conditions such as job strain, job control, job demands and social support at work [17,18]. There is also evidence of associations between CMD and relational and procedural justice [17,19], family-to-work and work-to-family conflicts [20] and workplace bullying [21,22].

Longitudinal studies examining the association between psychosocial working conditions and CMD are scarce, and report weaker associations than cross-sectional studies [17,23-25]. The longitudinal Maastricht Cohort Study examined the associations between several psychosocial working conditions and the onset of CMD [24]. Job demands, job control, social support at work, physical and emotional demands, job insecurity and conflicts at work were associated with onset one year later. However, none of these associations remained among the women following adjustment for previous CMD. Among the men, the associations remained with regard to all other psychosocial working conditions except job insecurity. Following replicating of the analysis among initially mentally healthy participants (excluding those with CMD at baseline) the results remained among the men, but only job demands, social support and emotional demands were associated with CMD onset among the women.

The evidence from longitudinal studies measuring only a few psychosocial working conditions is most consistent with regard to job demands and social support at work [7,9]. The Whitehall II study found an association among men between deterioration in CMD and high job demands, low job control and low social support from co-workers and supervisors after a one-to-four-year follow-up [26], whereas among women there was an association with job demands and social support at work. Adjusting for previous CMD attenuated the associations. When all the psychosocial working conditions were studied simultaneously in a longer follow-up period of four-to-eight years, only job demands and social support at work showed an association with deterioration among both men and women [27].

A Finnish study on hospital personnel identified associations between CMD and low procedural and relational justice, a high workload and hostility over a one-year follow-up [28]. Nevertheless, following adjustment for all psychosocial working conditions, the association remained only for procedural justice, workload and hostility. The Whitehall II study also found an association between relational justice and deterioration in CMD among both genders, even following adjustment for job demands, job control and social support at work [29].

A recent Finnish study found an association between workplace bullying and deterioration in CMD after six years [12]. Workplace bullying has also been associated with diagnosed depression after two years [30], and with the use of psychotropic medication after five years [31,32].

Studies examining the work-family interface have produced inconsistent results. Some report no association between work-to-family or family-to-work conflicts and deterioration in CMD measured during a period of three months [33] and one year [34]. However, researchers using a diagnostic interview (CIDI) to measure mental health have found associations between family-to-work [35] and work-to-family [11] conflicts and mental disorders in four-year and nine-year follow-ups.

In conclusion, we lack longitudinal studies on simultaneous associations between multiple psychosocial working conditions and common mental disorders. Most previous studies concentrate on the associations of a few psychosocial working conditions, and do not allow examination of the relative importance of various psychosocial factors. Improvement in CMD has not, to our knowledge, been examined previously, and covariates have only seldom been extensively taken into account. Enhancing knowledge about the conditions that most strongly affect deterioration or improvement in employee mental health could help in guiding the focus of practical interventions that promote employee wellbeing most effectively. It is also important to consider covariates such as limiting longstanding illnesses, which can affect people’s perceptions of their working environment and lead to exit from the work force due to selection in the labour market [36,37].

The aim of this study was to examine the associations between multiple psychosocial working conditions and subsequent change (improvement and deterioration) in CMD among middle-aged public-sector employees. We examined various psychosocial working conditions simultaneously, and considered several covariates including health behaviours, limiting longstanding illness, physical work, employment and marital status, and occupational position.

## Methods

### Data

The data were derived from the Helsinki Health Study baseline surveys conducted in 2001–2002 (*N* = 5819, response rate 66%) and a follow-up survey conducted in 2007 (*N* = 4805, response rate 83%). The respondents were employees of the City of Helsinki who had reached the age of 40, 45, 50, 50, 55 or 60 during 2001–2002. The majority (80%) were women, reflecting the proportion of women and men employed by the City of Helsinki and the municipal sector in general. According to the non-response analysis there were no significant differences in participation in the follow-up survey between those with and without CMD at baseline. Participation in the baseline study was somewhat more common among older employees, those in higher occupational classes, and those with worse health [38]. These differences remained in the follow up survey but were smaller than in the baseline survey and not fully consistent [39]. The analyses included participants with complete or median-substituted information (having answered at least 25% of the questions) on all the study variables (*N* = 4340).

### Common mental disorders

Common mental disorders were measured with the 12-item version of the General Health Questionnaire (GHQ-12), which is a well validated screening inventory for non-psychotic mental disorders and is widely used in population surveys [16,40]. The GHQ-12 items include questions on mood, emotions, self-worth and worries during the previous four weeks. The response alternatives are: “Better than usual”, “Same as usual”, “Worse than usual” and “Much worse than usual”. These are scored 0-0-1-1 and summed up following a standard procedure [40]. The total score ranges from 0–12. Validation studies recommend a cut-off point between scores 2 and 3 to indicate common mental disorders [40,41]. The GHQ inventory has shown high reliability and predictive validity against standardised clinical interviews [14,40]. It has also been shown to be a reliable and consistent instrument over relatively long time periods in general population samples [42].

### Psychosocial working conditions

The Framingham version of Karasek´s Job Content Questionnaire was used to measure job strain, which consists of the subscales job demands and job control [43,44]. Five items measure demands (Cronbach’s alpha α = .72) and nine items control (α = .82). Job control consists of decision authority and skill discretion. The demand and control scales were separately summed up and divided into quartiles. Job strain was measured according to Karasek’s [43] scale-construction system. First the demand and control scales, weighted according to Karasek’s [43] instructions, were dichotomized at the median, and the dimensions combined in four categories: “High job strain”, “Passive job”, “Active job”, and “Low job strain”. Missing values were replaced with item medians for those having answered at least four questions on demands and eight questions on control.

Moorman’s [45] inventory, which includes both procedural justice and relational justice, was used to measure organisational justice. Procedural justice consisted of four items (α = .87) measuring the degree to which the respondent perceives fair procedures to be present and used in the organisation. Relational justice measures the fairness of the supervisor’s behaviour, and similarly includes four items (α = .90). All the questions are answered on a five-point scale ranging from “1 = Strongly agree” to 5 = “Strongly disagree”. The item measures were separately summed up and divided into quartiles. If there was one missing value it was substituted by the median.

A modified version of Grzywacz & Marks’ [46] work-family interface inventory was used to measure work-to-family and family-to-work conflicts. Three items concerning negative work-to-family spill over (α = .83) assessed whether job responsibilities interfered with family life, and four items concerning family-to-work spill over (α = .93) assessed whether family responsibilities interfered with work. The response alternatives were 1 = “No disadvantage”, 2 = “Some disadvantage”, 3 = “Strong disadvantage”, 4 = “Not applicable/no family”. The items in both scales were summed up separately and divided into tertiles for the analysis. The rating of work-to-family conflicts varied from 3–9, classified as “No conflicts” (3), “Weak conflicts” (4–5) and “Strong conflicts” (6–9), whereas that of family-to-work conflicts varied from 4–12, classified as “No conflicts” (4), “Weak conflicts” (5–6) and “Strong conflicts” (7–12). The results for those with no family are not shown in the tables.

Social support was measured with a modified version of the six-item short form of the Social Support Questionnaire (SSQ6) [47]. Four items (α = .79) measured emotional, practical, relaxing and mind-lifting aspects of the social support perceived as available from partners, next of kin, friends, supervisors or co-workers, neighbors or other relatives. The respondent was instructed to choose one or more alternatives for each question. The sum was divided by the number of items and then split into thirds with tertiles as cut-off points. The three groups indicated the amount of perceived social support: low, average and high.

Workplace bullying was described as follows: “Mental violence or workplace bullying refers to the isolation of a member of the organisation, the underestimation of work performance, being threatened, being talked about behind one's back and other forms of pressure”. The question was: “Have you been subjected to such bullying behaviours?” and the response alternatives were: “No”, “Yes currently”, “Yes previously in this workplace”, “Yes previously in another workplace” and “Cannot say”. The two alternatives with regard to earlier bullying were collapsed.

The correlations between psychosocial working conditions ranged from weak to relatively strong (0.02 < *r* < 0.61). The strongest correlation was between procedural and relational justice. No collinearity was identified among the psychosocial working conditions.

### Covariates

We adjusted for covariates that have been found in previous studies to affect the association between psychosocial working conditions and common mental disorders [10,24]. All the covariates except employment status were obtained from the baseline questionnaire. Occupational class included four categories: professionals, semi-professionals, routine non-manual employees and manual workers. Marital status was classified into three categories: unmarried, cohabiting/married and divorced/widowed. Employment status was divided into two categories: those continuously employed and those not continuously employed (e.g., retired or unemployed) at follow-up. The dichotomized variables physical activity, alcohol consumption (in portions per week) and smoking status were used to measure health behaviours. The cut-off point for physical activity was 14 MET hours per week [48,49], and for alcohol consumption it was 16 portions for women and 24 portions for men. In terms of smoking status the division was between current smokers and non-smokers. Limiting long-term illness was considered in terms of sufferers and non-sufferers. With regard to physical effort, the respondents were asked how strenuous they considered their work, the resulting categories being light and strenuous.

### Statistical analysis

Given that the number of men was relatively small the analyses were conducted with pooled data for men and women. No statistically significant (p < 0.01) interactions were found between gender and psychosocial working conditions for follow-up CMD. In the case of deterioration, those with common mental disorders at baseline (GHQ score 3–12) were excluded from the analysis, and respectively in the case of improvement in CMD, those without (GHQ score 0–2) common mental disorders at baseline were excluded from the analysis. The respective comparison groups in the analysis comprised those with good psychosocial working conditions and those with poor psychosocial conditions. First, the age- and gender-adjusted prevalence of deterioration and improvement in CMD by each psychosocial factor was calculated. Second, logistic regression analysis was used to examine the associations between psychosocial working conditions and change in CMD, adjusting for the covariates. Age and gender were adjusted for in model 1. Additional adjustments for occupational class, marital and employment status, health behaviour, limiting longstanding illness and physical work were made in model 2, and still further adjustments for all psychosocial working conditions in model 3. The results are presented as odds ratios (OR) and their 95% confidence intervals (CI).

## Results

### The associations between psychosocial working conditions and deterioration and improvement in CMD

Common mental disorders remained stable between baseline and follow-up among 74% of the respondents: 63% consistently reported no CMD, and 11% consistently reported the presence of CMD. Furthermore, 26% reported a change between the two surveys: 13% a change for the worse and 13% a change for the better. Of the initially mentally healthy respondents (baseline GHQ-12 score < 3), 17% reported CMD at follow up, whereas 56% of those reporting CMD at baseline (GHQ-12 score > 2) reported none at follow-up.

Differences emerged between the categories of psychosocial working conditions in the association with deterioration in CMD (Table 1). Those reporting high job strain, and low relational and procedural justice were more likely to experience a deterioration than those reporting low job strain, and high relational and procedural justice. Moreover, respondents experiencing strong work-to-family or family-to-work conflicts and victims of current or earlier bullying at work were almost twice as likely to report a deterioration than those with no such experiences. In terms of social support there were only small differences between the categories in reporting deterioration in CMD.

**Table 1:** The prevalence of deterioration and improvement in common mental disorders (CMD) at follow-up by psychosocial working conditions at baseline^a^

	***N***	**Deterioration in CMD (GHQ > 2)**	***N***	**Improvement in CMD (GHQ < 3)**
All	3298	17 (15–18)	1042	56 (53–59)
Strain				
High	712	19 (17–22)	368	52 (47–57)
Passive job	891	16 (14–19)	242	54 (48–61)
Active job	761	18 (16–21)	280	58 (53–64)
Low	934	14 (12–17)	152	62 (54–70)
Relational justice				
Low	547	20 (17–23)	300	55 (49–60)
Moderate low	785	19 (17–22)	268	54 (48–60)
Moderate high	1097	15 (13–17)	261	60 (54–66)
High	869	15 (12–17)	213	55 (48–61)
Procedural justice				
Low	632	21 (18–23)	313	53 (48–59)
Moderate low	990	18 (16–21)	340	55 (50–61)
Moderate high	1023	15 (13–18)	249	60 (54–66)
High	653	13 (10–16)	140	55 (47–63)
Family-work conflicts				
Strong	386	24 (20–27)	314	51 (46–57)
Weak	978	19 (17–21)	336	57 (52–63)
No	1739	14 (12–16)	315	63 (57–68)
No family	195	18 (13–23)	77	40 (29–51)
Work-family conflicts				
Strong	848	24 (21–26)	536	54 (50–59)
Weak	1540	15 (13–17)	348	60 (55–66)
No	715	12 (9–15)	81	61 (50–72)
No family	195	18 (13–23)	77	40 (28–51)
Social support				
Low	1189	17 (15–19)	437	50 (45–55)
Average	920	18 (16–21)	303	60 (55–66)
High	1189	15 (13–18)	302	60 (54–65)
Workplace bullying				
Currently	111	23 (16–30)	92	45 (35–55)
Earlier	550	23 (20–26)	232	54 (48–60)
Cannot say	318	21 (17–25)	149	53 (45–60)
No	2319	14 (13–16)	569	59 (55–63)

^a^Age- and gender-adjusted percentages and 95% confidence intervals.

With regard to CMD improvement vis-à-vis deterioration, the differences between the categories of psychosocial working conditions were smaller (Table 1). There were only small differences in terms of job strain and both procedural and relational justice. Moreover, respondents experiencing no family-to-work or work-to-family conflicts, no bullying and high social support were more likely to report improvement in CMD as opposed to those experiencing strong family-to-work or work-to-family conflicts, current workplace bullying and low social support.

The associations between psychosocial working conditions and deterioration or improvement in CMD were further examined by means of logistic regression analysis (Tables 2 and 3). When adjusted for gender and age all psychosocial working conditions except social support were associated with a deterioration (Model 1). High job strain, having an active job, low and moderate procedural and relational justice, strong or weak family-to-work conflicts and current or earlier workplace bullying were associated with a deterioration in CMD. For example, those experiencing strong work-to-family conflicts at baseline were more likely (OR 2.36, CI 1.78-3.13) to show a deterioration at follow-up than those with no work-to-family conflicts. Adjusting additionally for occupational class, marital status and employment status, health behaviours, limiting longstanding illness and physical work only slightly affected these associations, except that the association between high job strain and deterioration was no longer statistically significant (Model 2). Simultaneous adjustment for all psychosocial working conditions clearly attenuated all the associations (Model 3), although they remained in the case of work-to-family and family-to-work conflicts and workplace bullying.

**Table 2:** The odds ratios for deterioration in common mental disorders at follow-up by psychosocial working conditions (N = 3298)^a^

	**Model 1**	**Model 2**	**Model 3**
Strain			
High	1.41 (1.08-1.83)	1.30 (0.99-1.72)	0.95 (0.71-1.28)
Passive job	1.16 (0.89-1.50)	1.07 (0.81-1.40)	0.93 (0.70-1.23)
Active job	1.34 (1.03-1.75)	1.28 (0.98-1.67)	0.98 (0.74-1.31)
Low	1.00	1.00	1.00
Relational justice			
Low	1.40 (1.06-1.86)	1.29 (0.97-1.72)	0.89 (0.62-1.28)
Moderate low	1.39 (1.07-1.80)	1.36 (1.05-1.77)	1.07 (0.79-1.45)
Moderate high	0.98 (0.76-1.26)	0.98 (0.76-1.26)	0.88 (0.67-1.16)
High	1.00	1.00	1.00
Procedural justice			
Low	1.74 (1.28-2.35)	1.65 (1.21-2.24)	1.35 (0.92-1.99)
Moderate low	1.51 (1.14-2.00)	1.47 (1.11-1.96)	1.30 (0.93-1.82)
Moderate high	1.20 (0.90-1.61)	1.24 (0.93-1.66)	1.20 (0.88-1.65)
High	1.00	1.00	1.00
Family-to-work conflicts			
Strong	1.93 (1.46-2.55)	1.93 (1.45-2.57)	1.41 (1.04-1.91)
Weak	1.43 (1.16-1.78)	1.49 (1.19-1.86)	1.26 (1.00-1.59)
No	1.00	1.00	1.00
Work-to-family conflicts			
Strong	2.36 (1.78-3.13)	2.55 (1.89-3.45)	1.99 (1.42-2.78)
Weak	1.32 (1.00-1.73)	1.39 (1.05-1.83)	1.22 (0.91-1.63)
No	1.00	1.00	1.00
Social support			
Low	1.14 (0.91-1.42)	1.07 (0.85-1.35)	1.04 (0.82-1.32)
Average	1.23 (0.98-1.55)	1.15 (0.91-1.46)	1.13 (0.89-1.44)
High	1.00	1.00	1.00
Bullying			
Currently	1.74 (1.08-2.79)	1.57 (0.97-2.53)	1.25 (0.75-2.08)
Earlier	1.75 (1.38-2.21)	1.60 (1.27-2.04)	1.40 (1.09-1.79)
Cannot say	1.55 (1.15-2.09)	1.45 (1.07-1.96)	1.31 (0.96-1.78)
No	1.00	1.00	1.00

^a^Analysis were conducted for the pooled data for men and women, those with CMD at baseline (GHQ >2) being excluded from the analysis.

Model 1: adjusted for age and gender.

Model 2: adjusted for model 1 + SEP + marital status + employment status + health behaviour (smoking, alcohol consumption, physical activity) + limiting longstanding illness + physical work.

Model 3: adjusted for model 2 and psychosocial working conditions.

**Table 3:** The odds ratios for improvement in common mental disorders at follow-up by psychosocial working conditions (N = 1042)^a^

	**Model 1**	**Model 2**	**Model 3**
Strain			
Low	1.49 (1.01-2.20)	1.43 (0.95-2.14)	1.39 (0.89-2.17)
Active job	1.28 (0.94-1.76)	1.24 (0.89-1.75)	1.26 (0.88-1.81)
Passive job	1.07 (0.77-1.48)	1.06 (0.76-1.49)	1.02 (0.72-1.46)
High	1.00	1.00	1.00
Relational justice			
High	0.97 (0.68-1.39)	0.91 (0.63-1.31)	0.74 (0.47-1.16)
Moderate high	1.22 (0.87-1.71)	1.15 (0.82-1.63)	0.91 (0.61-1.37)
Moderate low	0.93 (0.66-1.29)	0.93 (0.66-1.30)	0.85 (0.59-1.23)
Low	1.00	1.00	1.00
Procedural justice			
High	1.03 (0.68-1.54)	0.97 (0.64-1.47)	0.87 (0.53-1.45)
Moderate high	1.30 (0.93-1.83)	1.22 (0.86-1.74)	1.13 (0.75-1.71)
Moderate low	1.07 (0.79-1.46)	0.99 (0.72-1.36)	0.97 (0.69-1.39)
Low	1.00	1.00	1.00
Family-to-work conflicts			
No	1.62 (1.17-2.26)	1.69 (1.20-2.37)	1.65 (1.16-2.34)
Weak	1.30 (0.95-1.77)	1.33 (0.97-1.82)	1.29 (0.93-1.79)
Strong	1.00	1.00	1.00
Work-to-family conflicts			
No	1.30 (0.80-2.11)	1.42 (0.86-2.35)	1.27 (0.74-2.17)
Weak	1.28 (0.97-1.68)	1.30 (0.97-1.73)	1.19 (0.88-1.62)
Strong	1.00	1.00	1.00
Social support			
High	1.48 (1.09-2.00)	1.40 (1.02-1.93)	1.34 (0.96-1.86)
Average	1.52 (1.13-2.06)	1.51 (1.11-2.05)	1.47 (1.07-2.01)
Low	1.00	1.00	1.00
Bullying			
No	1.72 (1.10-2.70)	1.58 (1.00-2.49)	1.58 (0.97-2.58)
Cannot say	1.32 (0.78-2.23)	1.22 (0.71-2.08)	1.28 (0.73-2.23)
Earlier	1.40 (0.86-2.29)	1.35 (0.82-2.22)	1.50 (0.90-2.52)
Currently	1.00	1.00	1.00

^a^Analysis were conducted for the pooled data for men and women, N = 1042, those without CMD at baseline (GHQ < 3) being excluded from the analysis.

Model 1: adjusted for age and gender.

Model 2: adjusted for model 1 + SEP + marital status + employment status + health behaviour (smoking, alcohol consumption, physical activity) + limiting longstanding illness + physical work.

Model 3: adjusted for model 2 and psychosocial working conditions.

The associations between psychosocial working conditions and improvement in CMD were examined next (Table 3). Low job strain, no family-to-work conflicts, high and average social support and not being bullied at work were associated with an improvement in CMD following adjustment for age and gender (Model 1). For example, those not being bullied were more likely (OR 1.72, CI 1.10-2.70) to show an improvement than those currently being bullied. Adjusting additionally for the covariates in Model 2 had only slight effects on these associations. However, job strain was no longer associated with an improvement. Simultaneous adjustments for all psychosocial factors attenuated the associations, and in the fully adjusted model 3 no family-to-work conflicts and average social support remained associated with improvement in CMD.

## Discussion

### Main findings

The aim of this study was to examine the associations between multiple psychosocial working conditions and both deterioration and improvement in common mental disorders, and to find out whether several covariates and other psychosocial working conditions affected these associations. The first main finding was that person-centred psychosocial working conditions that also captured conditions outside work, such as work-to-family and family-to-work conflicts, contributed more to deterioration and improvement in CMD than environmental conditions. Fewer conditions were related to improvement than to deterioration. Second, taking into account socio-demographic factors and health-related covariates had only minor effects. However, considering all the psychosocial factors simultaneously attenuated the associations. In the fully adjusted models work-to-family and family-to-work conflicts and workplace bullying showed the strongest associations with deterioration in CMD, whereas family-to-work conflicts and social support showed the strongest associations with an improvement.

### The results of this study in the light of previous studies

Only a few previous longitudinal studies have examined the work-family interface and workplace bullying in conjunction with several other psychosocial working conditions. Studies concentrating on work-family interface show inconsistent results. In accordance with our results, a Finnish study [50] found an association between strong family-to-work conflicts and a deterioration in CMD in a one-year follow-up, but no associations between work-to-family or family-to-work conflicts and deterioration in CMD were found in two earlier studies [33,34]. The effects of family-to-work and work-to-family conflicts found in our study could be understood with regard to the importance of both family and work values among women and the number of dual-earner couples in the sample. It has been suggested that work-to-family and family-to-work conflicts are intensified when either work or family roles are salient to the person's self-concept [51]. The number of women participating in working life in OECD countries is increasing, and work-related values are becoming as important as family-related values [52]. These changes in roles and values may be reflected in higher levels of stress experienced by dual-earner couples trying to fulfil both work and family expectations.

We found an association between both current and earlier workplace bullying and a deterioration in CMD, which is in accordance with the results of previous longitudinal studies [12,30]. Bullying has been associated with various health-related outcomes: on the social level it is related to stigmatization, exclusion and rejection [53], on the psychological level it causes nervous symptoms, apathy, depression and anxiety [54], and on the psychosomatic level it has been associated with somatic and mental symptoms [30,31]. Moreover, it has been suggested that workplace bullying may represent a traumatic event [55]. Individuals exposed to long-term and severe bullying have manifested symptoms resembling those of post-traumatic stress disorder [56]. Moreover, bullying and CMD may also have reciprocal associations [30] due to stigmatization: mentally ill people may have problems coping with their daily tasks and cooperating with colleagues, which in turn may make them targets of bullying [57]. Furthermore, continuous bullying increases the risk of poor mental and even physical health [58].

According to the results of the analyses in which we simultaneously included all the psychosocial working conditions, social support was associated only with an improvement in CMD: this differs from findings of previous studies suggesting that low levels of social support at work are related to deterioration in CMD [24,26,27]. However, there is a lack of research on the association between social support and improvements in CMD. The discrepancy in the results may be attributable to the different measurements used in the studies. Our measure may have captured the emotional form of social support better in that the respondents were asked to indicate one or more persons from whom they received support, which may have been the people closest to them. Previous studies have used measures focusing on social support at work, which may better capture the instrumental/practical form of support.

Relational and procedural justice were associated with deterioration in CMD when the covariates were considered, which is in line with previous findings [28,29]. However, these associations were no longer statistically significant when all the psychosocial working conditions were taken into account. A similar attenuation was found with job strain, which in previous studies remained associated with deterioration in CMD when other psychosocial conditions were taken into account [26,27]. We examined the associations between CMD and both job control and job demands separately in sensitivity analyses, but the results remained the same: the association was no longer significant following adjustment for the covariates. Simultaneous examination of organisational justice and job strain is lacking in previous studies.

There are several possible mechanisms that may explain the stronger associations between CMD and both work-family interface and workplace bullying than other psychosocial working conditions. First, psychosocial working conditions may be partly overlapping, which attenuates the association when they are studied simultaneously [18,59]. For example, workplace bullying is likely to overlap with other aspects of the social context such as organisational justice, social support and job strain. Our sensitivity analyses supported this assumption in that workplace bullying attenuated most of the associations between both relational and procedural justice and deterioration in CMD.

Second, psychosocial working conditions may have spill-over effects [37] when two contexts interfering with each other boost each other’s effects [60]. For example, having family-to-work conflicts is related to having work-to-family conflicts, and vice versa [61], which our sensitivity analyses confirmed. It may be that the effects of person-centred conditions continued to influence the employees at home, and therefore these conditions had more adverse effects on CMD than other psychosocial working conditions.

Third, the importance of psychosocial working conditions may differ according to the occupational environments and occupations under study [62]. One third of our cohort worked in a caring occupation at baseline and was middle-aged. Emotional pressure may be more relevant to common mental disorders than tempo- and speed-related variables in healthcare settings [7], which may partly explain the non-existent associations with job strain we found. Job strain has been criticized for not measuring psychological strain [63], and it may therefore not be fully suitable as a measure for our study population. The participants may also have elderly parents to take care of, which in turn may explain the strong effects of the work-family interface.

Future studies would benefit from using a broad framework of psychosocial working conditions in different work environments. It would also be worth taking into account potential confounders such as personal factors and critical life events, and examining the mediating and moderating effects. Neuroticism has shown an independent effect on depression and attribution style in previous studies, for example, whereas stressful life events such as interpersonal conflicts have been found to contribute to psychological disorders [60].

### Methodological considerations

The strengths of this study include the large prospective sample, the reliable CMD measure and the wide variety of psychosocial working conditions. We were able to control for several covariates and to take into account previous common mental disorders in separate analyses of those with and without CMD at baseline.

However, there are problems with multiple testing and caution is needed when drawing conclusions from the analyses of this study. A high number of tests increases the risk for type I error [64]. As we included several independent variables and adjusted for several covariates this may lead to false positive findings. As sensitivity analyses we conducted type 3 tests to examine the significance of each partial effect with all the other effects in the model. However, this did not affect the original results. To avoid type I error, our sensitivity analyses also used Bonferroni adjustment that divides the critical *P* value (α) by the number of comparisons being made. As expected the statistical significance was lost in some of the associations. However, it should be also taken into account that the use of adjustments is controversial [65] and may lead to different kind of problems such as increase the risk of type II error [64] and lower statistical power [65].

Taking into account previous mental health has attenuated or even eliminated the association between psychosocial working conditions and mental health in previous studies [24,66]. However, even excluding participants with CMD at baseline does not rule out the effect of negative affectivity, which may be reflected in the way employees report their psychosocial working conditions [9]. Nevertheless, previous studies have shown that it has only minor to moderate effects on the association between psychosocial working conditions and CMD [21,37]. The use of independent and more objective measures of working conditions as well as more objective outcomes in addition to self-reports is warranted and may produce more reliable results [9,37].

The rather long follow-up period may also cause limitations as the effects of psychosocial working conditions and CMD may have fluctuated over the follow-up. It was not possible to obtain information on the duration and the intensity of psychosocial working conditions, neither was it possible to take into account pre-baseline CMD. However, previous studies report that psychosocial working conditions remain quite stable over time [34,36]. It is possible that employees exposed to poorer psychosocial working conditions at baseline were more likely to move to different jobs, which may have affected the results. The study sample consisted of employees working for the City of Helsinki, thus given that psychosocial working conditions vary in different occupations and work environments, the results cannot be directly generalised to other age groups or employment sectors. The measures of both common mental disorders and psychosocial working conditions were based on self-reports, and should be critically assessed for potential bias: personal traits such as negative affectivity may influence the reports, for example. Critical life events may have affected CMD but could not be taken into account. Although the GHQ is widely used and has been shown to be a reliable measure of mental health, it does not capture mental disorders such as psychotic and dependency disorders [1]. Furthermore, dichotomized measuring does not capture changes above or below the score of three in the GHQ, although as a threshold score it has been recommended and validated against clinical measures of mental health [14,15,40,41]. Nevertheless, self-reported CMD may be more useful than diagnostic interviews or psychiatric diagnosis for screening mental-health problems in the occupational context because it captures also minor problems. It can also be used in wider populations when clinical interviews would be non-practical and expensive.

## Conclusions

The complex associations among several psychosocial working conditions should be better understood in order to promote employee mental health by focusing on the most relevant conditions. Using a broad psychosocial framework our study suggests that family-to-work conflicts, work-to-family conflicts and workplace bullying likely increases the risk of CMD, whereas social support and having no family-to-work conflicts are likely to be supportive factors among those recovering from such disorders. Preventing the onset of CMD and improving mental health among employees who currently show symptoms would be beneficial in terms of helping employees and employers to manage the compelling demands of work and family, preventing workplace bullying and promoting social support both at work and at home. Measures for reducing family-work conflicts might focus on enhancing self-efficacy and coping strategies, and providing more flexible working schedules [61]. Early identification of workplace bullying [30] and supporting cooperative problem and conflict solving [67] might equally help to reduce workplace bullying and to ensure a supportive social environment.

## Abbreviations

CMD: Common mental disorders; GHQ: General health questionnaire.

## Competing interests

The authors declare that they have no competing interests.

## Authors’ contributions

HL conducted the statistical analyses, interpreted the results and drafted the manuscript. PS, JL, EL and OR contributed to designing the study, interpreting results and drafting the manuscript. All the authors critically reviewed the manuscript and approved the final version.

## Pre-publication history

The pre-publication history for this paper can be accessed here:

http://www.biomedcentral.com/1471-2458/14/588/prepub
